# Differential gene expression profiling and machine learning-based discovery of key genetic markers in VTE and CKD

**DOI:** 10.3389/fimmu.2025.1654673

**Published:** 2025-10-22

**Authors:** Hui Li, Cai Lin, Junjie Kuang

**Affiliations:** Emergency Department, Huizhou First Hospital, Huizhou, Guangdong, China

**Keywords:** VTE (venous thromboembolism), CKD (chronic kidney disease), differential gene expression, machine learning algorithms, diagnostic nomogram

## Abstract

**Introduction:**

Venous thromboembolism (VTE) and chronic kidney disease (CKD) are multifactorial disorders characterized by complex genetic and molecular mechanisms. However, their shared genetic signatures and potential interrelations remain poorly understood. This study aimed to identify key genes and molecular pathways linking VTE and CKD through comprehensive transcriptomic and machine learning analyses.

**Methods:**

Gene expression profiles from patients with VTE and CKD, along with corresponding controls, were analyzed to identify differentially expressed genes (DEGs). Functional enrichment analyses were performed using Gene Ontology (GO) and Kyoto Encyclopedia of Genes and Genomes (KEGG) pathways. The intersection of DEGs between VTE and CKD was used for feature selection via three machine learning algorithms: Least Absolute Shrinkage and Selection Operator (LASSO), Support Vector Machine–Recursive Feature Elimination (SVM-RFE), and Random Forest (RF). A diagnostic nomogram was constructed based on key genes, followed by receiver operating characteristic (ROC) curve analysis, gene set enrichment analysis (GSEA), and immune infiltration assessment. Validation was performed using independent datasets (GSE37171 and GSE48000) and single-cell RNA sequencing data.

**Results:**

A total of 637 DEGs (413 upregulated and 224 downregulated) were identified in VTE patients, and 671 DEGs (99 upregulated and 572 downregulated) were identified in CKD patients. Enrichment analyses revealed that VTE DEGs were primarily involved in cytoplasmic translation, immune activation, and oxidative phosphorylation, while CKD DEGs were enriched in muscle contraction regulation, ATPase activity, and vascular smooth muscle contraction. Twenty-three overlapping DEGs were found between CKD and VTE, including CCNL2, HNRNPA0, PI4KA, FOS, and HBD. Machine learning analyses identified HNRNPA0 and PI4KA as the most robust feature genes, both exhibiting excellent diagnostic performance (AUC = 1.000). A diagnostic nomogram based on these genes showed strong predictive accuracy and calibration. GSEA and immune infiltration analyses revealed their involvement in immune-related and metabolic pathways. Validation in external datasets confirmed significantly lower expression of HNRNPA0 and PI4KA in CKD samples. Single-cell RNA sequencing further delineated their expression across 11 cellular clusters corresponding to eight major cell types.

**Discussion:**

This study identifies HNRNPA0 and PI4KA as key genes shared between VTE and CKD, providing new insights into their genetic and immunological links. The diagnostic model based on these genes offers a promising tool for CKD prediction and highlights potential targets for future mechanistic and therapeutic investigations.

## 1 Introduction

Venous thromboembolism (VTE) and chronic kidney disease (CKD) are two complex and multifactorial diseases that have profound impacts on global public health (1, 2). Both diseases, individually, have been the focus of extensive research over the past decades due to their high prevalence, significant morbidity, and mortality (2, 3). However, the precise molecular mechanisms underlying their pathogenesis remain elusive.

VTE, which encompasses both deep vein thrombosis (DVT) and pulmonary embolism (PE), is a critical condition arising from the formation of blood clots in the deep veins, particularly of the legs (4, 5). It poses a significant health threat, with millions of individuals affected worldwide. If untreated, these clots can break off and travel to the lungs, resulting in a potentially fatal pulmonary embolism (6). The intricate nature of VTE, with its multifactorial origin encompassing genetic, environmental, and behavioral factors, makes its diagnosis and treatment challenging (7, 8). Although genetic markers like Factor V Leiden mutation are well-known for their association with VTE (9), a comprehensive genetic landscape detailing the interaction of multiple genes remains to be uncovered.

Chronic kidney disease is another grave health concern that affects a significant portion of the global population (10). CKD gradually leads to a loss of kidney function over time and can culminate in kidney failure (11). Like VTE, the development and progression of CKD are influenced by a myriad of factors, both genetic and environmental (12, 13). While the genetic predispositions of certain populations to CKD are known, the vast network of genetic interactions and their impact on disease progression and severity remain an area rife for exploration.

Given the complexities associated with VTE and CKD, a molecular-level understanding is paramount. The advent of advanced genomic technologies, especially next-generation sequencing, has offered a profound insight into the genetic underpinnings of various diseases (14). By utilizing such cutting-edge techniques, researchers have the tools necessary to decipher the intricate gene networks and pathways associated with these diseases. A holistic understanding can guide clinicians in early detection, prognosis assessment, and personalized treatment approaches, offering a paradigm shift from the traditional one-size-fits-all model.

The intersection of genetics and computational methods has ushered in the era of genomics-driven personalized medicine (15). Machine learning, a subfield of artificial intelligence, has demonstrated immense potential in decoding the vast amounts of genomic data (16). By employing algorithms that can ‘learn’ from and make decisions based on data, machine learning can facilitate the identification of genetic markers and predictive modeling for diseases like VTE and CKD. This convergence of genomics and computational methodologies offers a promise to elucidate previously unrecognized genetic interactions and networks integral to the pathophysiology of these conditions.

Considering the abovementioned background and the potential of an integrated approach, this study was conceived. Our primary aim was to delve deep into the genetic landscape of VTE and CKD, identify differentially expressed genes (DEGs), and employ machine learning algorithms to pinpoint key genetic markers. By shedding light on these markers, we hoped to pave the way for a better understanding of disease mechanisms, early detection strategies, and possibly, more targeted therapeutic interventions.

## 2 Methods

### 2.1 Data retrieval

Gene expression data associated with CKD and VTE were obtained from the Gene Expression Omnibus (GEO) database. For CKD, the GSE66494 dataset, based on the GPL6480 platform, included placenta samples from 48 CKD patients and 5 healthy controls, and the GSE37171 dataset, based on GPL570, contained 75 CKD samples and 40 healthy controls, which was used to validate diagnostic efficiency and the robustness of simulated gene expression results. For VTE, the GSE19151 dataset, based on the GPL571 platform, comprised 70 VTE patient and 63 healthy control blood samples, and the GSE48000 dataset, based on GPL10558, including 15 VTE and 11 healthy control samples, served as an independent validation set. Additionally, single-cell RNA sequencing data for CKD (GSE198621) incorporating samples from 3 CKD cases and 3 normal controls were utilized to validate the expression levels of key genes at the single-cell level. All datasets were retrieved in accordance with the GEO repository protocols. Prior to analysis, expression data were processed for quality control and normalized, and batch effect correction was performed using the ComBat method to reduce heterogeneity arising from different platforms and experimental conditions.

### 2.2 Identification and enrichment analysis of differentially expressed genes

Differentially expressed genes (DEGs) were identified using the “limma” R package for both CKD and VTE datasets. For the GSE19151 dataset, genes with |log2 fold change (FC)| ≥ 0.5 and p < 0.05 were considered differentially expressed, while for the GSE66494 dataset, the threshold was set at |log2FC| ≥ 1 and p < 0.05. The different thresholds were selected considering the sample sizes and variability in each dataset, consistent with previously published studies. To minimize potential bias due to platform differences, all datasets were subjected to batch effect correction prior to DEG analysis. Functional enrichment analysis was conducted using the “clusterProfiler” package, including Gene Ontology (GO) and Kyoto Encyclopedia of Genes and Genomes (KEGG) pathway enrichment. Pathways with p < 0.05 were considered significantly enriched. For validation at the single-cell level, key DEGs were mapped to the CKD single-cell dataset to confirm their expression patterns across different cell types.

### 2.3 Identification of crosstalk genes

The crosstalk genes for the two diseases were obtained by hybridizing the DEGs of CKD with those of VTE. To reduce the high false discovery rate, these genes were further screened using the Wilcoxon test (p < 0.05) between CKD and control samples, and a similar analysis was carried out between VTE and control samples.

### 2.4 Establishment of the diagnostic model

To identify key gene features for the diagnostic model, we utilized three machine learning algorithms: LASSO (Least Absolute Shrinkage and Selection Operator), Random Forest, and SVM-RFE (Support Vector Machine Recursive Feature Elimination). For LASSO, we employed Cox proportional hazards regression using the “glmnet” R package. Cross-validation was applied to optimize the regularization parameter (λ) via 10-fold cross-validation, where the λ value that minimized the mean cross-validation error was selected. The coxnet option was used to perform survival analysis, with 100 iterations to ensure model stability.

For Random Forest, we used the randomForest R package to rank the importance of CKD-related marker genes. The model was configured with 500 trees, which is the default setting, and the number of variables at each split was set to the square root of the total number of features. To minimize overfitting, a 5-fold cross-validation was performed, and genes with an importance score greater than 0.8, as determined by the mean decrease in Gini index, were considered for further analysis.

SVM-RFE was implemented using the e1071 R package with a linear kernel. The cost parameter (C) was set to 1 to balance the trade-off between maximizing the margin and minimizing classification errors. The algorithm was run for 10 iterations to identify the most relevant features, with genes ranked based on the average performance across all iterations. A 10-fold cross-validation approach was used to assess the optimal number of features to retain, focusing on accuracy and error rates.

In the final step, a hybrid approach was adopted, combining the results from LASSO, Random Forest, and SVM-RFE. This consensus approach helped to identify a set of robust and reliable gene features for further validation. The diagnostic performance of these marker genes was evaluated using Receiver Operating Characteristic (ROC) curve analysis, and the Area Under the Curve (AUC) was calculated to assess their ability to distinguish CKD samples. To validate the diagnostic potential, the expression profiles of the selected genes were analyzed in external datasets, including GSE37171, GSE19151, and GSE48000.

### 2.5 Modeling and validation of PE diagnostic nomogram

The diagnostic nomogram was created using the “rms” R package. Patient scores were calculated based on the expression of individual core genes, with the total risk score defined as the sum of risk scores for all individual genes. The dCA curve, calibration curve, and ROC curve were utilized to evaluate the diagnostic value of the nomogram for CKD.

### 2.6 Enrichment analysis of key genes and immune cell infiltration

GSEA was used to analyze the biological function of the key genes. Based on the gene sets of 28 immune-related cells, the ssGSEA algorithm from the R package “GSVA” was employed to assess the immune activity for each sample. Differences in immune infiltration between the CKD and VTE groups were analyzed. Additionally, the correlation between immune infiltration levels and the expression of key genes in the two diseases was investigated.

### 2.7 scRNA-seq analysis

For single-cell characterization studies, the scRNA-seq dataset GSE198621 was analyzed using the standard protocol of “Seurat”. Cells with fewer than 100 genes, more than 5000 total genes, and mitochondrial gene content exceeding 20% were further filtered out. The R package “Harmony” was employed to mitigate batch effects between samples. The “FindVariableFeatures” function was used to identify the top 2000 variably expressed genes. Cells were annotated using “SingleR”, and the expression of key genes in different cell types was subsequently validated.

### 2.8 Quantitative real-time PCR validation of key genes

Peripheral blood samples were collected from 15 patients with clinically diagnosed CKD (stage 3–4) and 15 age- and sex-matched healthy controls at Huizhou First People’s Hospital, following approval by the institutional Ethics Committee and acquisition of written informed consent from all participants.

Peripheral blood mononuclear cells (PBMCs) were isolated from whole blood using Ficoll-Paque density gradient centrifugation according to standard protocols. Total RNA was extracted from PBMCs using the FastPure Cell/Tissue Total RNA Isolation Kit (Vazyme, China) following the manufacturer’s instructions. RNA concentration and purity were assessed by NanoDrop spectrophotometry. Reverse transcription was performed using the ReverTra Ace qPCR RT Master Mix with gDNA Remover (Toyobo, Japan) to synthesize complementary DNA (cDNA).

qRT-PCR was conducted using SYBR Premix Ex Taq II (Takara, Japan) on a real-time PCR system. The thermocycling conditions were as follows: initial denaturation at 95 °C for 10 minutes, followed by 45 cycles of 95 °C for 5 seconds and 60 °C for 30 seconds. The housekeeping gene GAPDH was used as the internal control. Primer sequences are listed in **Supplementary File 1**.

### 2.9 Statistical analysis

All statistical analyses were performed using R (version 4.3.2) and GraphPad Prism 9.0. Differential expression was determined with the “limma” package, and p values were adjusted using the Benjamini–Hochberg method. Comparisons between two groups were analyzed using Student’s t-test or the Wilcoxon rank-sum test as appropriate. Correlations were assessed by Spearman’s method. Diagnostic performance was evaluated by ROC curve analysis, and qRT-PCR results were calculated using the 2^–ΔΔCt method. A two-tailed p < 0.05 was considered statistically significant. In figures, significance is indicated as follows: p < 0.05 (*), p < 0.01 (**), p < 0.001 (***), and p < 0.0001 (****).

## 3 Results

### 3.1 Identification of differentially expressed genes

**Figure 1:**
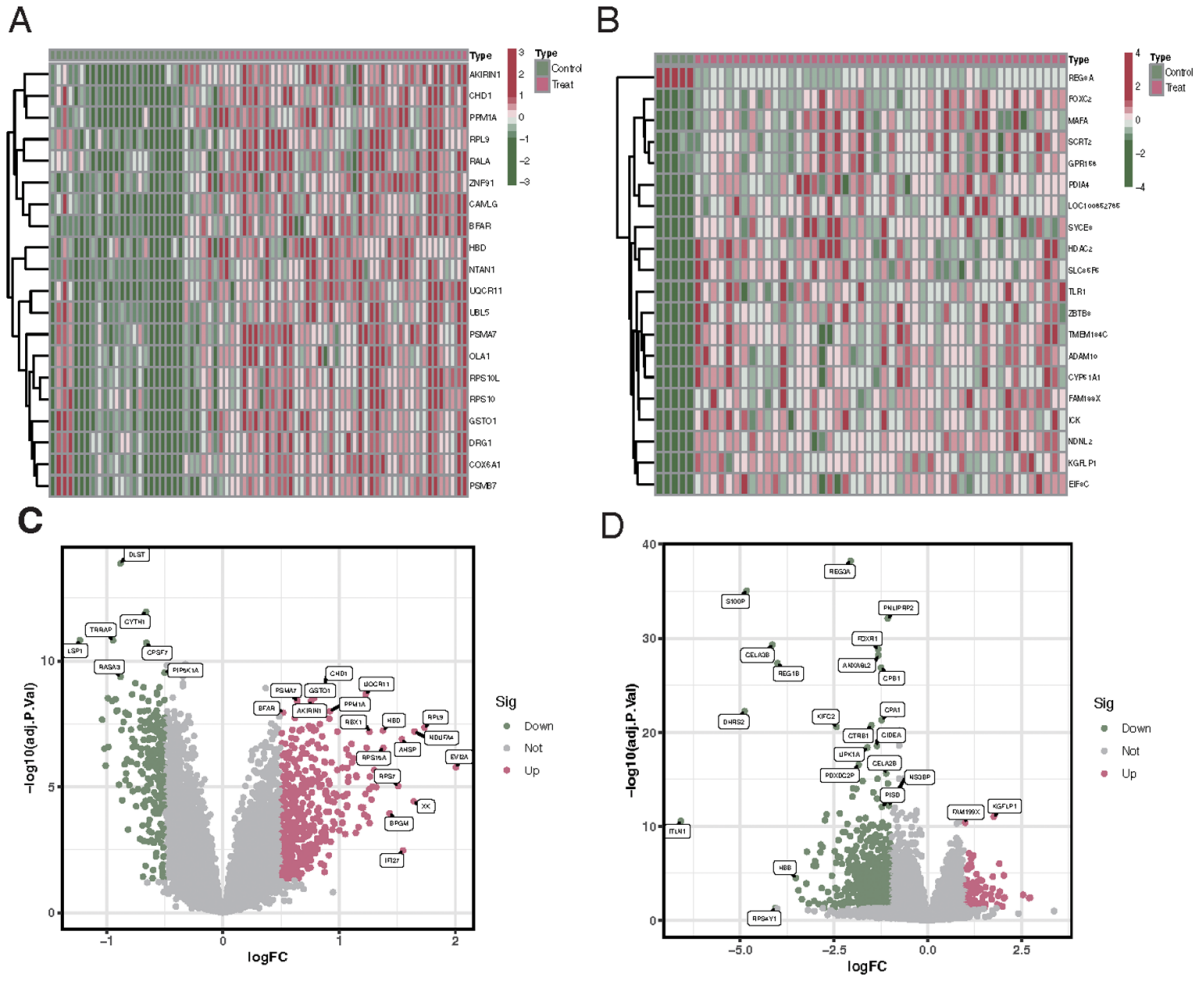
Differential gene expression profiles in VTE and CKD patients compared to controls. **(A)** Upset plot showing the number of downregulated (224) and upregulated (413) genes in VTE patients versus control. **(B)** Upset plot illustrating the number of downregulated (572) and upregulated (99) genes in CKD patients versus control. **(C)** Heatmap representing the expression profiles of the differentially expressed genes in VTE patients compared to controls. Red indicates upregulated genes, and blue indicates downregulated genes. **(D)** Heatmap showcasing the expression profiles of the differentially expressed genes in CKD patients compared to controls. Red denotes upregulated genes, while blue indicates downregulated genes.

Differential analysis revealed that between VTE patients and the control group, there were 637 differentially expressed genes, of which 224 were downregulated and 413 were upregulated (**Figures 1A, C**). Analysis of differences between CKD patients and the control group showed 671 differentially expressed genes, including 572 downregulated genes and 99 upregulated genes (**Figures 1B, D**).

### 3.2 Enrichment analysis of differentially expressed genes

**Figure 2:**
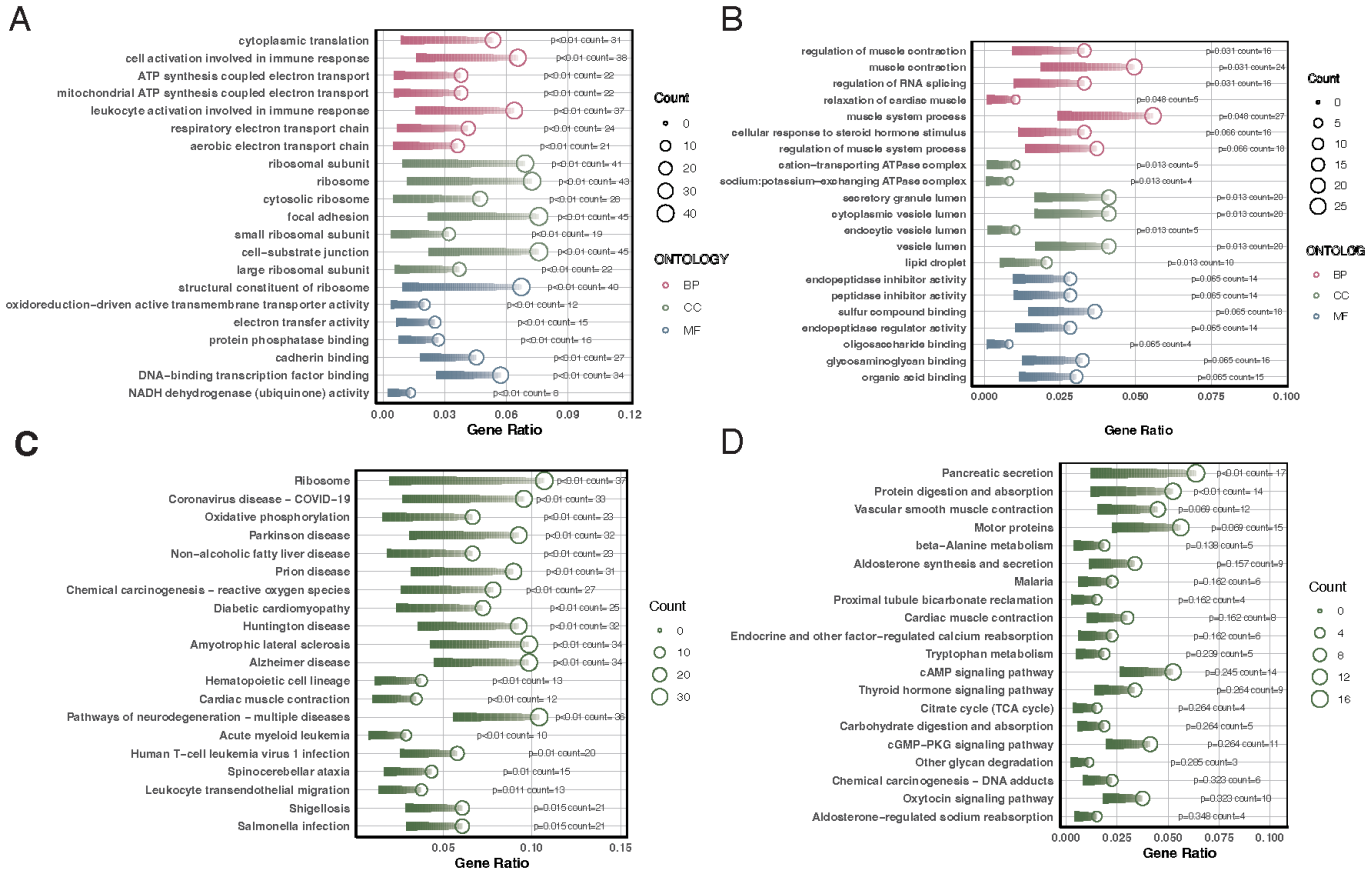
GO and KEGG enrichment analyses of differentially expressed genes in VTE and CKD. **(A)** Bar chart illustrating the top GO enrichment terms for differentially expressed genes in VTE patients, highlighting cytoplasmic translation, cell activation in the immune response, and ribosomal subunits. **(B)** Bar chart demonstrating the top GO enrichment terms for differentially expressed genes in CKD patients, emphasizing the regulation of muscle contraction, cation-transporting ATPase complex, and endopeptidase inhibitor activity. **(C)** Bar chart depicting the top KEGG pathways enriched in VTE differentially expressed genes, with a focus on oxidative phosphorylation, reactive oxygen species related to chemical carcinogenesis, and 2019 coronavirus disease signaling pathways. **(D)** Bar chart revealing the top KEGG pathways enriched in CKD differentially expressed genes, highlighting pancreatic secretion, protein digestion and absorption, and vascular smooth muscle contraction.

The biological functions of differentially expressed genes in different diseases were explored through GO and KEGG enrichment analyses. Differentially expressed genes in VTE patients were mainly enriched in cytoplasmic translation, cell activation involved in the immune response, and ribosomal subunits (**Figure 2A**). KEGG enrichment analysis showed that VTE differentially expressed genes were mainly enriched in oxidative phosphorylation, reactive oxygen species associated with chemical carcinogenesis, and signaling pathways of the 2019 coronavirus disease (**Figure 2C**). Differentially expressed genes in CKD patients were primarily enriched in regulation of muscle contraction, cation-transporting ATPase complex, and endopeptidase inhibitor activity (**Figure 2B**). KEGG enrichment analysis showed that CKD differentially expressed genes were primarily enriched in signaling pathways such as pancreatic secretion, protein digestion and absorption, and vascular smooth muscle contraction (**Figure 2D**).

### 3.3 Identification of crosstalk genes

**Figure 3:**
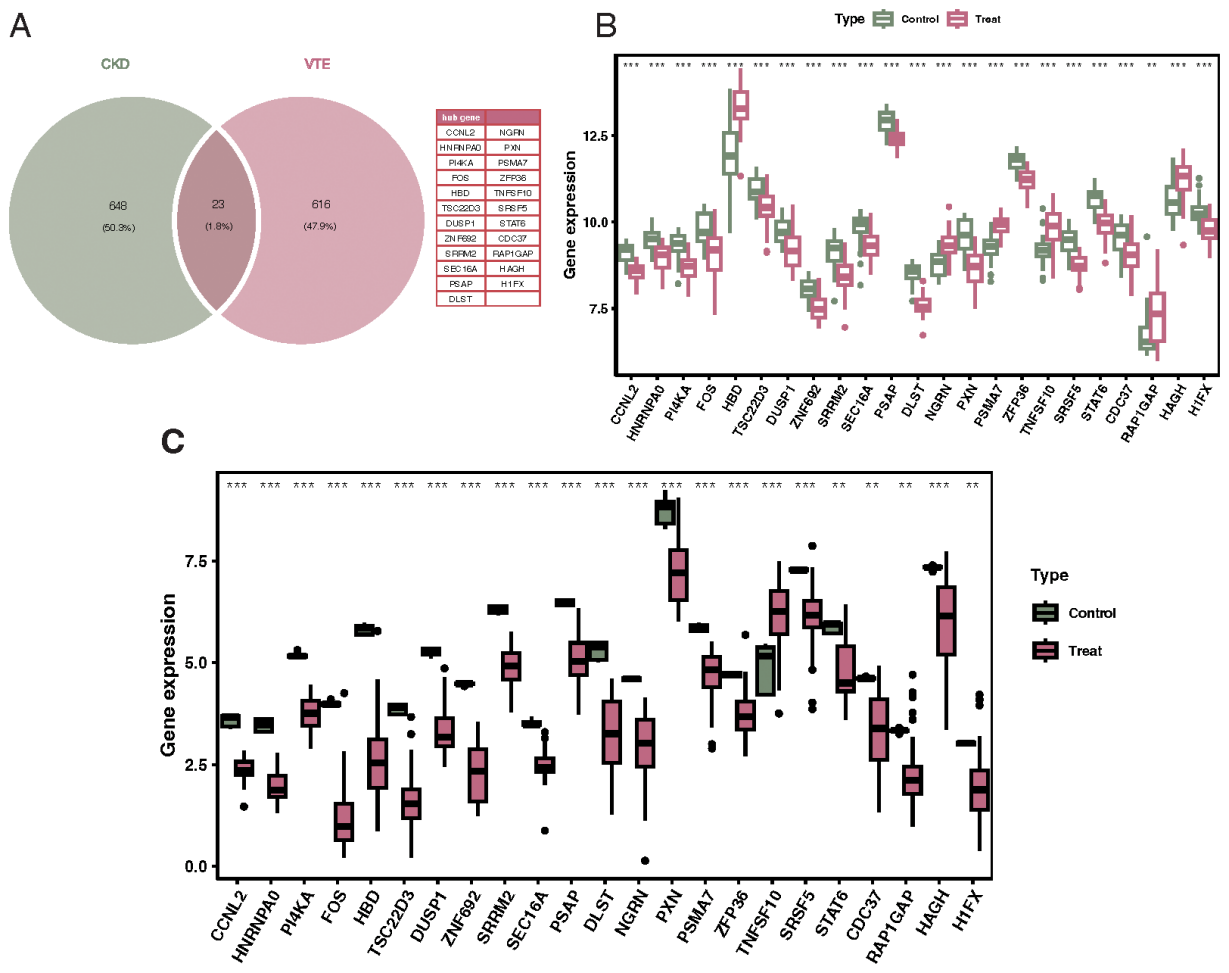
Identification of crosstalk genes differentially expressed in both CKD and VTE. **(A)** Venn diagram showcasing the overlap of 23 genes that are differentially expressed in both CKD and VTE diseases, with notable genes labeled. **(B)** Bar chart displaying the expression levels of the 23 overlapping genes in CKD patients versus controls, indicating their differential expression significance. **(C)** Bar chart illustrating the expression levels of the 23 overlapping genes in VTE patients versus controls, emphasizing their differential expression significance. **p < 0.01, ***p < 0.001.

Venn diagram results identified 23 overlapping genes differentially expressed in both CKD and VTE diseases, including CCNL2, HNRNPA0, PI4KA, FOS, HBD, TSC22D3, DUSP1, and ZNF692 (**Figure 3A**). Results from the Wilcoxon test indicated that these 23 genes were significantly differentially expressed in both CKD and VTE diseases (**Figures 3B, C**).

### 3.4 Feature gene selection using LASSO, random forest, and SVM-RFE algorithms

**Figure 4:**
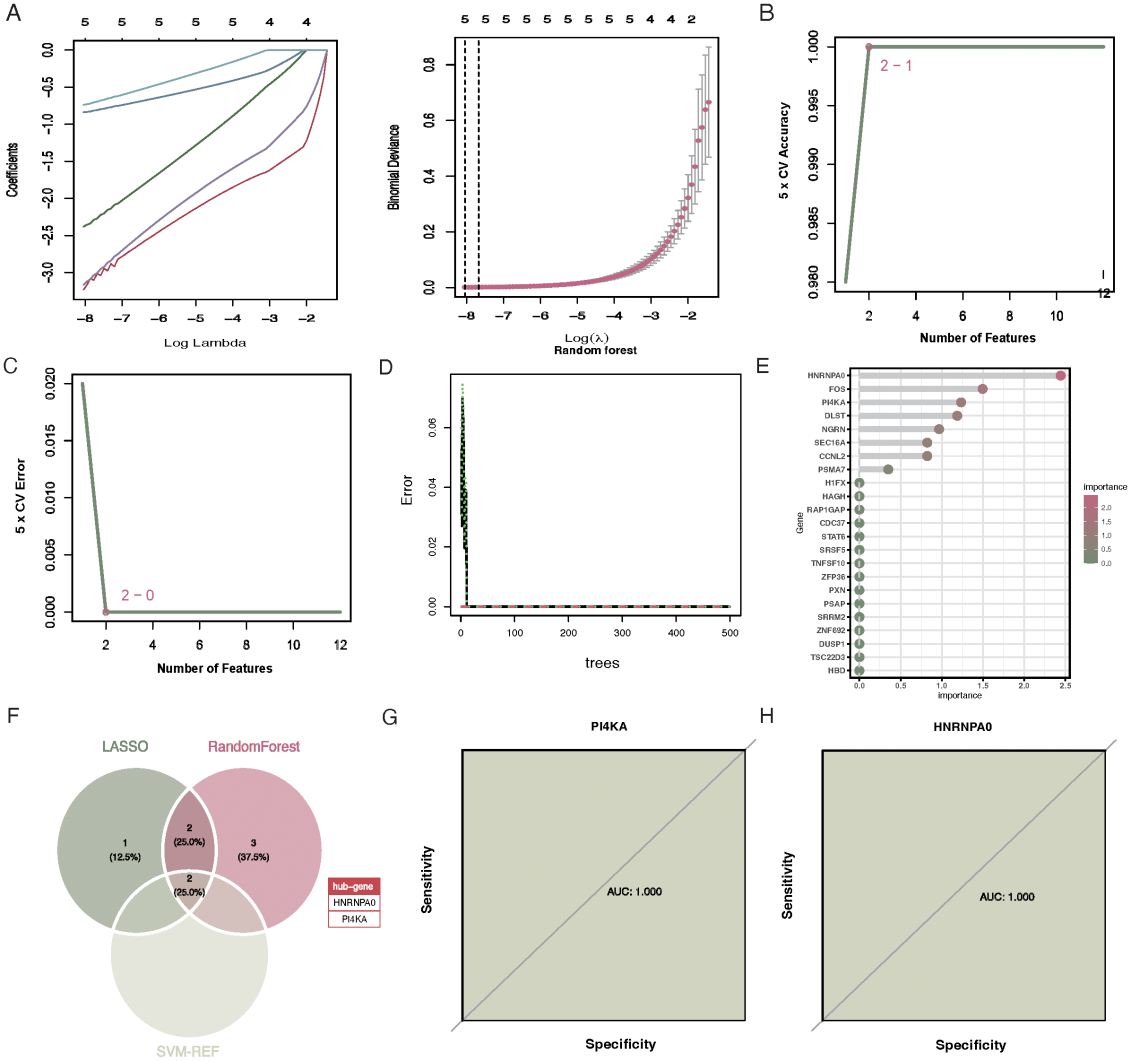
Selection of feature genes via LASSO, SVM-RFE, and Random Forest algorithms. **(A)** Tuning of the regularization penalty in LASSO with a 10-fold cross-validation. The optimal lambda value is indicated by the vertical line. **(B)** Feature ranking in SVM-RFE, highlighting HNRNPA0 and PI4KA as the two most significant features. **(C)** Variable importance plot for the SVM-RFE algorithm, emphasizing the significance of the top features. **(D)** Variable importance plot for the Random Forest algorithm. The y-axis represents the genes, and the x-axis denotes their significance. Genes with significance greater than 0.8 are highlighted. **(E)** Bar chart showcasing the seven significant genes selected by the Random Forest algorithm. **(F)** Venn diagram illustrating the overlap between the feature genes selected by the three algorithms, identifying HNRNPA0 and PI4KA as the key genes. **(G)** ROC curve for HNRNPA0, indicating an AUC value of 1.000. **(H)** ROC curve for PI4KA, demonstrating an AUC value of 1.000.

Upon performing 10-fold cross-validation, the optimal lambda for the LASSO algorithm was determined to be 0.002. The penalty parameter (l) corresponding to the minimal partial likelihood deviance was selected, yielding 5 feature genes: CCNL2, HNRNPA0, PI4KA, FOS, and HBD (**Figures 4A**). In contrast, for SVM-RFE, the model classifier with the highest accuracy and the lowest error had 2 features: HNRNPA0 and PI4KA (**Figures 4B, C**). The random forest algorithm selected 7 genes with significance greater than 0.8, including HNRNPA0, FOS, PI4KA, DLST, NGRN, CCNL2, and SEC16A (**Figures 4D, E**). Based on the aforementioned three machine learning algorithms, two key genes were selected: HNRNPA0 and PI4KA (**Figure 4F**). The AUC for the key genes HNRNPA0 and PI4KA were both 1.000 (**Figures 4G, H**).

### 3.5 Construction and testing of the CKD prediction nomogram based on feature genes

**Figure 5:**
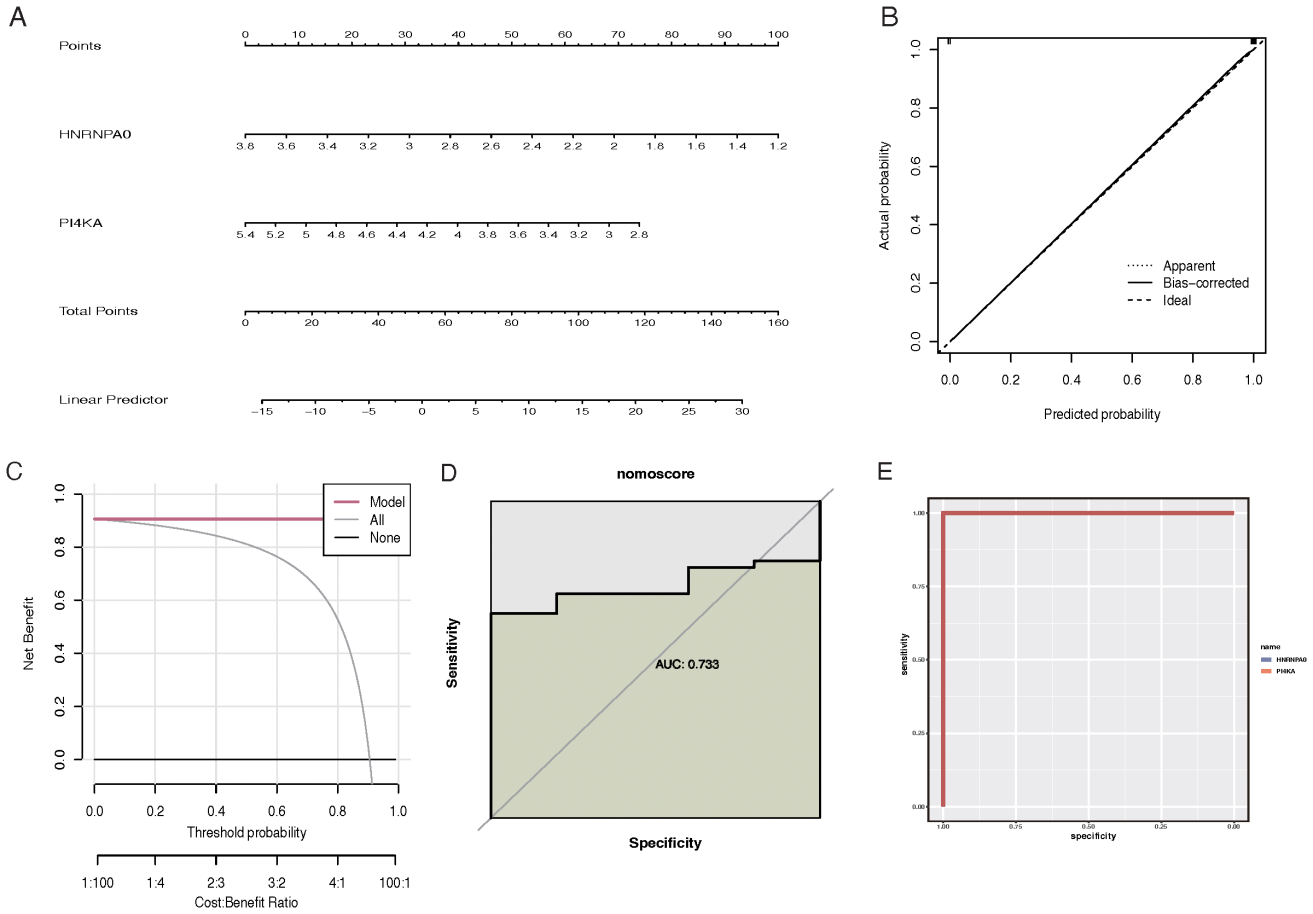
Construction and evaluation of the CKD prediction nomogram based on feature genes. **(A)** Nomogram for CKD risk prediction. Feature genes HNRNPA0 and PI4KA are used as predictive factors with points assigned for each gene expression level. The total points are then converted to a probability of CKD diagnosis. **(B)** Calibration curve for the CKD prediction nomogram. The y-axis represents the observed CKD risk, and the x-axis represents the predicted CKD risk. A 45-degree diagonal line indicates perfect calibration. **(C)** Decision curve analysis (DCA) for the CKD prediction nomogram. The y-axis represents the net benefit, and the x-axis denotes the threshold probability. The nomogram model’s curve is compared to the treat-all-patients and treat-none curves. **(D)** ROC curve analysis for the gene-based model, highlighting the performance of feature genes in predicting CKD. **(E)** ROC curve analysis for the CKD prediction nomogram, showcasing its diagnostic accuracy.

The “rms” R package was used to construct a nomogram model for RM diagnosis based on the feature genes (HNRNPA0 and PI4KA) (**Figure 5A**). Calibration curves indicated a minimal discrepancy between the predicted CKD risk and the actual CKD risk, suggesting that the nomogram model has high accuracy (**Figure 5B**). Decision curve analysis (DCA) demonstrated that CKD patients could benefit from the nomogram model (**Figure 5C**). The validity of this model was also confirmed by gene and nomogram ROC curve analyses (**Figures 5D, E**).

### 3.6 GSEA enrichment analysis and key gene immune cell infiltration

**Figure 6:**
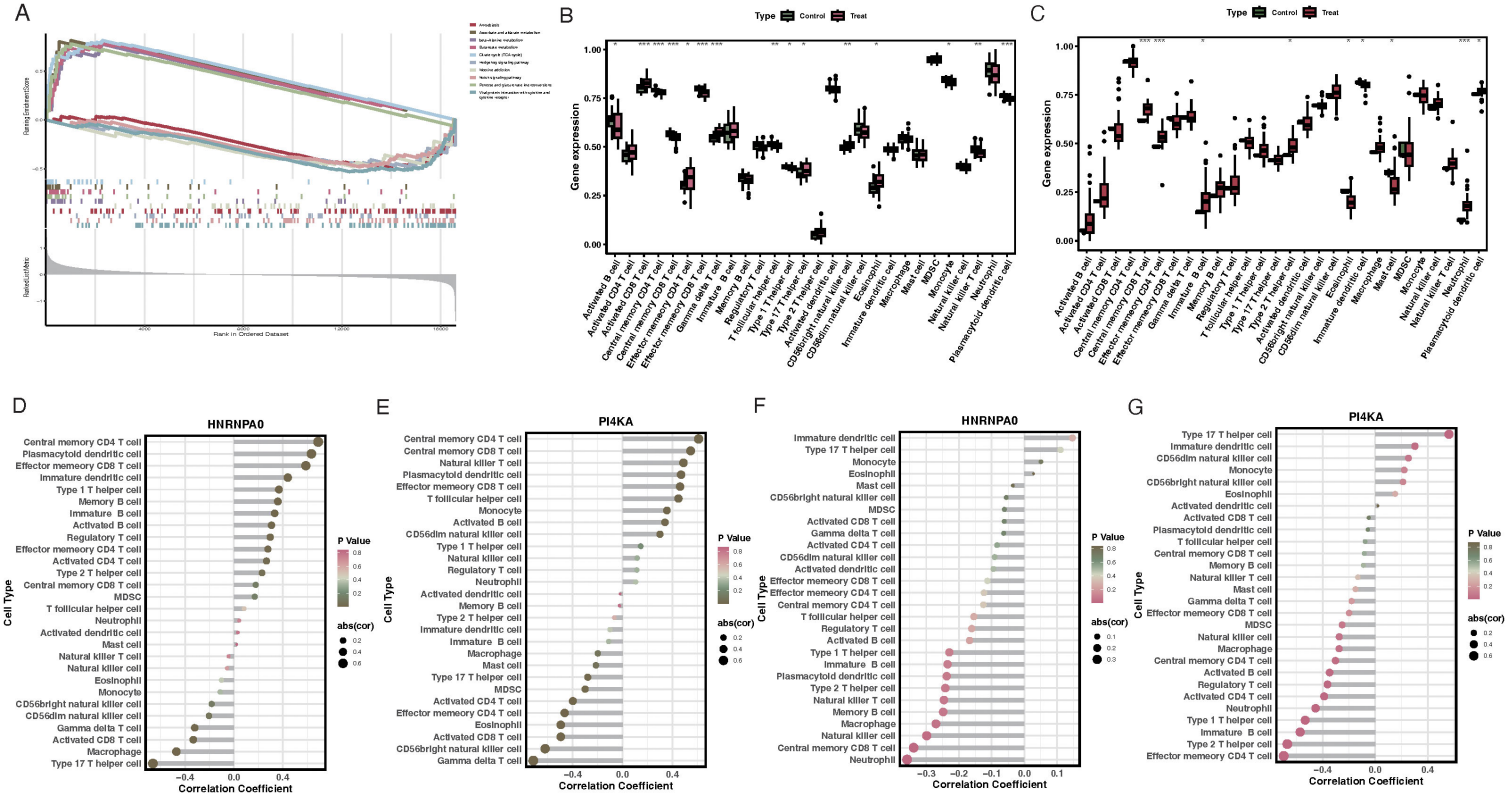
GSEA enrichment analysis and immune cell infiltration associated with key genes. **(A)** Enrichment plots for HNRNPA0 and PI4KA, emphasizing its association with pathways like Amoebiasis and Ascorbate and aldarate metabolism. **(B)** ssGSEA analysis of immune cell infiltration in VTE patients, highlighting the relative infiltration levels of specific immune cells. **(C)** ssGSEA analysis of immune cell infiltration in CKD patients from GSE66494 dataset. **(D)** Correlation heatmap illustrating the relationship between HNRNPA0 expression and infiltration of various immune cell types in the GSE19151 dataset. **(E)** Correlation heatmap depicting the association between PI4KA expression and infiltration of different immune cell types in the GSE19151 dataset. **(F)** Correlation analysis between HNRNPA0 and specific immune cell types in the GSE66494 dataset. **(G)** Correlation analysis between PI4KA and certain immune cell types in the GSE66494 dataset. *p < 0.05, **p < 0.01, ***p < 0.001.

GSEA results suggested that HNRNPA0 was primarily involved in pathways like Amoebiasis, Ascorbate and aldarate metabolism (**Figure 6A**). PI4KA was mainly enriched in pathways like Ascorbate and aldarate metabolism and Butanoate metabolism (**Figure 6A**). Results from ssGSEA for immune cell infiltration revealed that VTE patients exhibited higher infiltration levels of activated CD8 T cells, effector memory CD4 T cells, CD56bright natural killer cells, and eosinophils, while having lower infiltration of activated B cells, central memory CD4 T cells, and plasmacytoid dendritic cell-like cells (**Figure 6B**). For GSE19151, the immune cell infiltration analysis revealed that HNRNPA0 was significantly associated with 15 immune cell types, positively correlating with cell types such as Central memory CD4 T cell, Plasmacytoid dendritic cell, Effector memory CD8 T cell, and negatively correlating with 4 immune cell types including Type 17 T helper cell, Macrophage (**Figure 6D**). PI4KA showed positive correlations with 9 immune cell types and negative correlations with 8 cell types including Type 17 T helper cell, Effector memory CD4 T cell (**Figure 6E**). For GSE66494, ssGSEA analysis indicated a higher infiltration of Type 2 T helper cells and Immature B cells in CKD patients (**Figure 6C**). Within GSE66494, the correlation analysis between immune cells and key genes showed that HNRNPA0 negatively correlated with Central memory CD8 T cell, Neutrophil (**Figure 6F**). PI4KA exhibited a positive correlation with cell types including Type 17 T helper cell and negative correlations with cell types such as Effector memory CD4 T cell, Type 2 T helper cell (**Figure 6G**).

### 3.7 Validation of key genes

**Figure 7:**
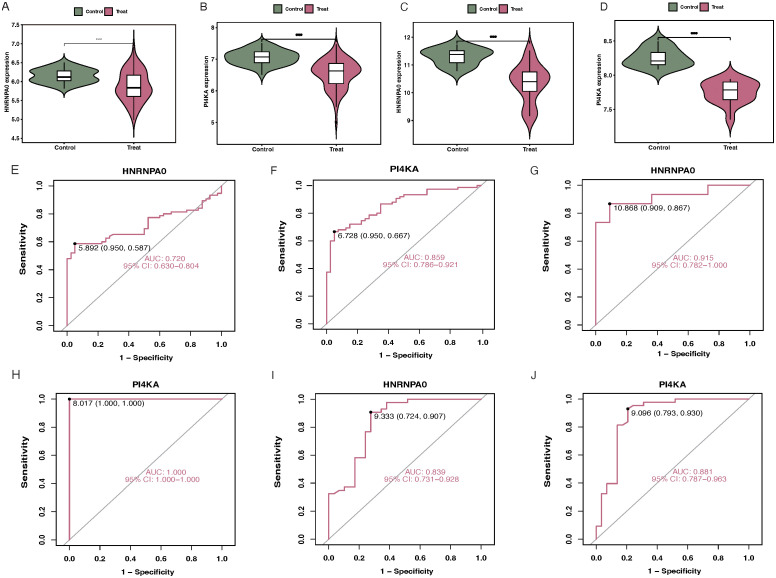
Validation of key genes in different datasets. **(A, B)** Boxplots depicting the expression levels of HNRNPA0 and PI4KA in CKD patients compared to controls in the GSE37171 dataset. **(C, D)** Differential expression analysis of HNRNPA0 and PI4KA in CKD patients vs. controls in the GSE48000 dataset. **(E, F)** ROC curve analyses for HNRNPA0 and PI4KA in the GSE37171 dataset, highlighting their respective AUC values. **(G, H)** ROC curve analyses for HNRNPA0 and PI4KA in the GSE48000 dataset, emphasizing their diagnostic accuracy. **(I, J)** ROC curve analyses for HNRNPA0 and PI4KA in the GSE19151 dataset, showcasing their AUC values.

For the GSE37171 dataset, the expression levels of HNRNPA0 and PI4KA in CKD patients were lower than in normal samples (**Figures 7A, B**). The AUC for HNRNPA0 and PI4KA were 0.720 and 0.859, respectively (**Figures 7E, F**). Similarly, differential expression analysis of the key genes in the GSE48000 dataset produced consistent results (**Figures 7C, D**). Furthermore, the AUC for HNRNPA0 and PI4KA were 0.985 and 1.000, respectively (**Figures 7G, H**). ROC curve analysis for key genes in GSE19151 showed that the AUC for HNRNPA0 and PI4KA were 0.839 and 0.881, respectively (**Figures 7I, J**).

### 3.8 Expression of key genes at the single-cell level

**Figure 8:**
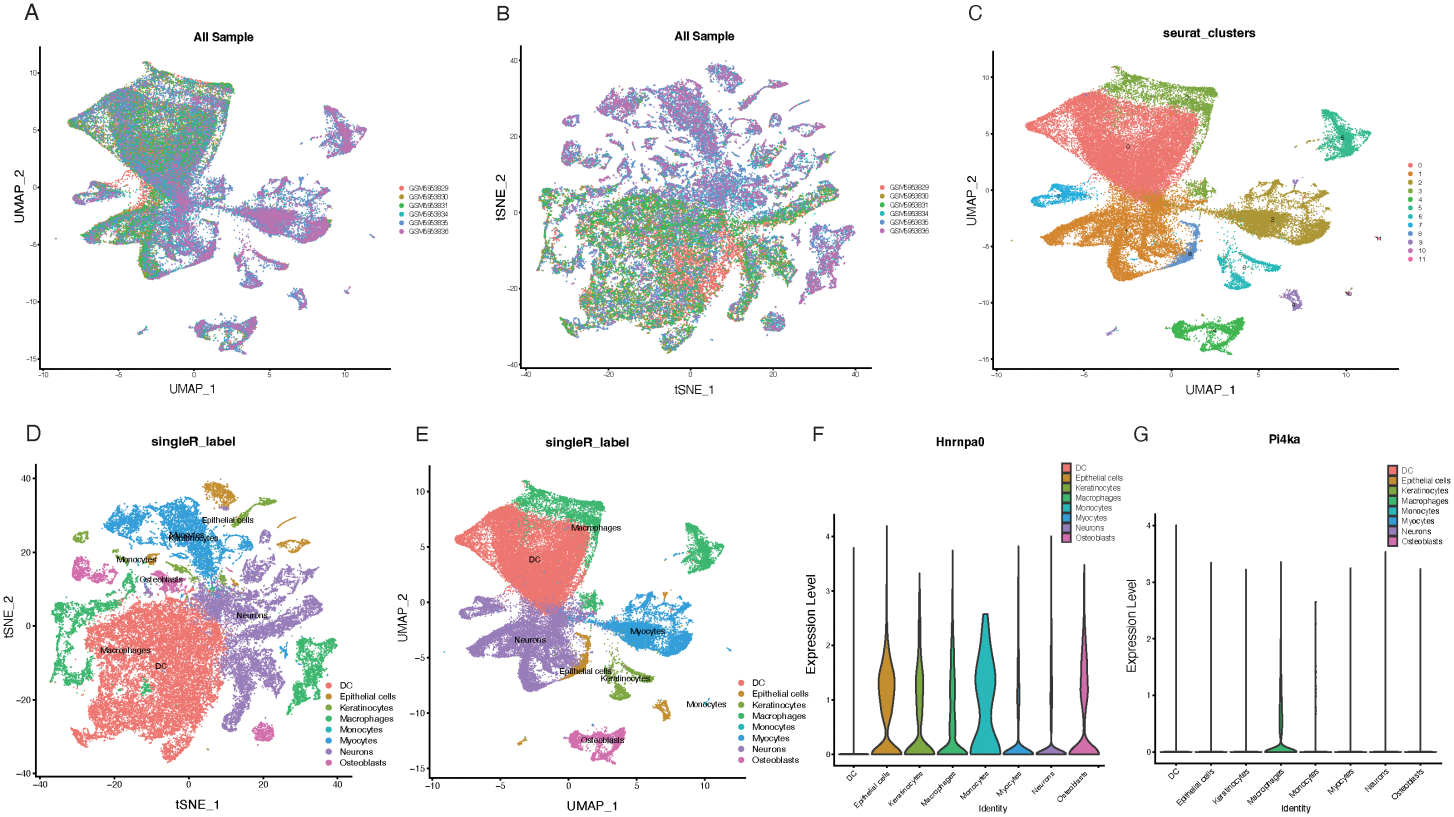
Single-cell analysis of key gene expression in CKD samples. **(A, B)** UMAP plots visualizing gene expression profiles from control samples (25,449 cells) and CKD samples (29,463 cells). **(C)** UMAP visualization of 11 identified cell clusters based on gene expression profiles. **(D)** Annotation of the 11 cell clusters, leading to the determination of eight distinct cell types. **(E)** UMAP plot displaying the distribution of the eight annotated cell types. **(F)** Expression heatmap showcasing the expression levels of HNRNPA0 across the eight identified cell types. **(G)** Expression heatmap illustrating the expression levels of PI4KA across the eight determined cell types.

Following the data filtering and integration described in the methods, we obtained gene expression profiles for 25,449 cells from control samples and 29,463 cells from CKD samples (**Figures 8A, B**). Clustering all cells, we identified 11 cell clusters (**Figure 8C**). Annotation and visualization of the 11 cell clusters resulted in eight cell types, such as Epithelial cells and Macrophages (**Figures 8D, E**). Finally, the expression of key genes across different cell types was displayed (**Figures 8F, G**).

### 3.9 qRT-PCR validation of key gene expression in CKD patients

**Figure 9:**
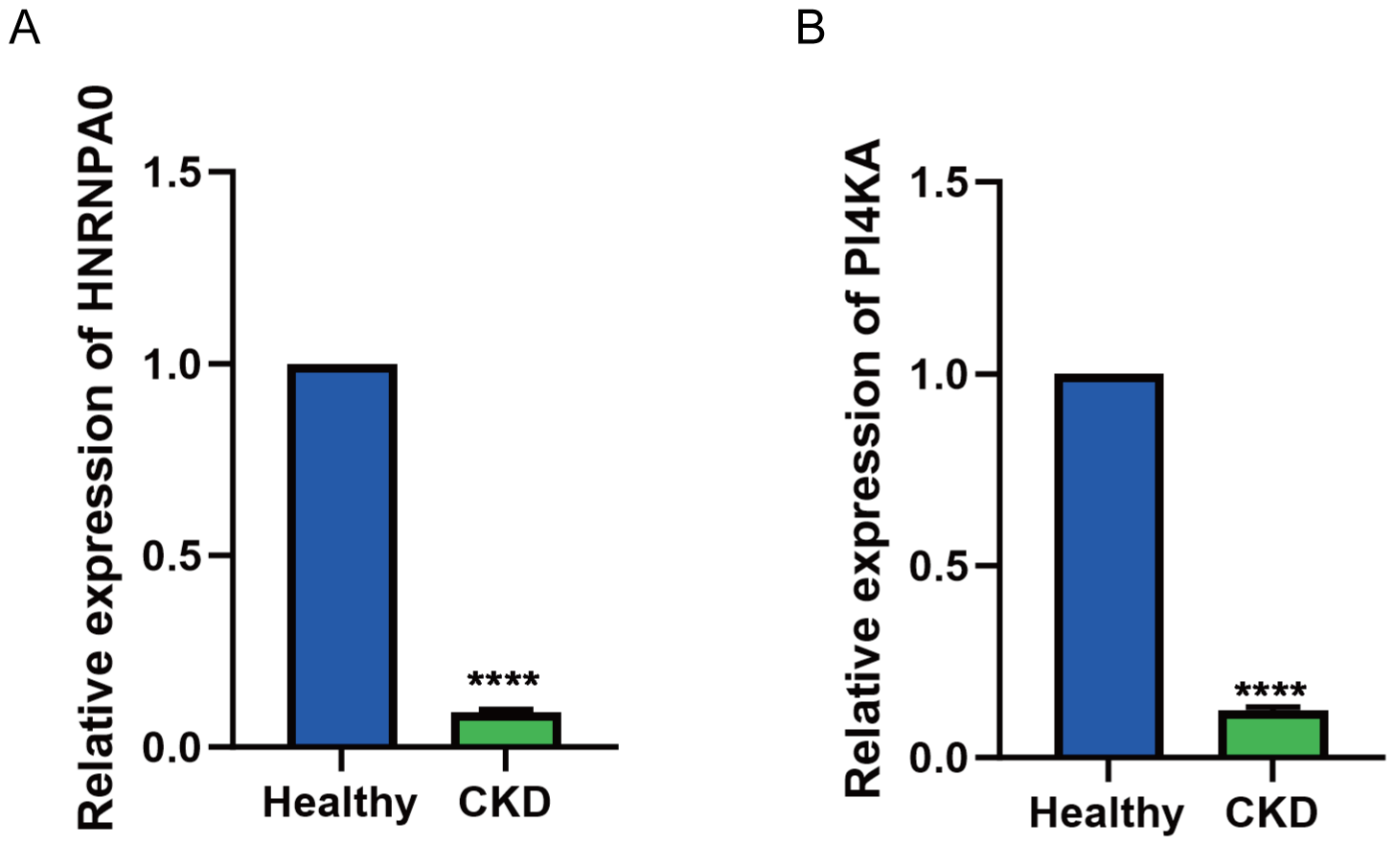
Validation of HNRNPA0 and PI4KA expression in PBMCs from CKD patients by qRT-PCR. **(A, B)** Bar graph showing the relative mRNA expression levels of HNRNPA0 and PI4KA in PBMCs from CKD patients (n = 15) and healthy controls (n = 15). ****p < 0.0001.

To validate the expression of key genes identified from the transcriptomic and single-cell analyses, we performed qRT-PCR on PBMCs collected from 15 CKD patients and 15 healthy controls. Consistent with the multi-omics and single-cell findings, both HNRNPA0 and PI4KA exhibited significantly lower mRNA expression levels in CKD patients compared to healthy individuals (**Figures 9A, B**).

## 4 Discussion

In this study, we employed a multi-omics approach integrating transcriptomic analysis, machine learning, and single-cell RNA sequencing to identify and validate key genes involved in VTE and CKD. Through comprehensive differential expression analysis, functional enrichment, cross-disease gene screening, and feature gene selection via LASSO, SVM-RFE, and random forest algorithms, we identified HNRNPA0 and PI4KA as robust biomarkers shared by both diseases. These genes were further validated in independent datasets and confirmed to be significantly upregulated in CKD patients using qRT-PCR. Our findings highlight the potential involvement of these genes in the pathogenesis of CKD and suggest their utility as predictive biomarkers.

HNRNPA0, a member of the heterogeneous nuclear ribonucleoprotein (hnRNP) family, has been previously characterized as a downstream effector of the p38/MK2 checkpoint kinase pathway, operating independently of p53 to regulate cell cycle arrest and mRNA stability via AU-rich elements (AREs) (17–19). Notably, HNRNPA0 stabilizes Gadd45α and p27(Kip1) mRNAs upon DNA damage, contributing to chemotherapy resistance in lung cancer and to hematopoietic lineage fate decisions in murine models through post-transcriptional regulation of ARE-containing transcripts (20). These functions suggest a broader role in inflammatory signaling and cellular stress responses—key hallmarks in both CKD progression and thrombotic predisposition.

In our study, HNRNPA0 was significantly upregulated in both CKD and VTE samples, and its expression strongly correlated with immune cell infiltration, particularly effector/memory T cells and plasmacytoid dendritic cells, while showing negative associations with macrophages and Th17 cells. These observations support its putative role as a modulator of immune homeostasis. Interestingly, HNRNPA0 was found to be highly expressed in macrophages, which aligns with findings from recent studies indicating that HNRNPA0 can regulate mRNA stability and subcellular distribution in immune cells. Specifically, HNRNPA0 has been shown to bind to the 3’-UTR of CCR2, a receptor crucial for macrophage migration, influencing both CCR2 mRNA stability and its subcellular localization (21). In line with recent findings in neurodegenerative diseases where HNRNPA0 forms insoluble aggregates with tau protein, its dysregulation may also reflect broader RNA-processing disturbances under chronic inflammatory conditions (22, 23). Furthermore, its altered expression in aging-associated hematopoiesis and myeloid differentiation implies potential involvement in immune senescence and immune dysregulation, both prominent features of advanced CKD and pro-thrombotic states (24, 25).

PI4KA is a lipid kinase involved in the generation of phosphatidylinositol 4-phosphate (PI4P), a precursor of PIP2, which is central to multiple cell signaling cascades including AKT, ERK, and calcium pathways (26–28). Structurally, PI4KA exists as part of a heterotrimeric complex with TTC7 and FAM126, which facilitates its recruitment to the plasma membrane via EFR3A/B, enabling spatial control of signaling (29). Disruption of this complex has been shown to impair PI4KA localization and activity, with consequences for membrane dynamics, autophagy, and energy metabolism.

Our results indicated that PI4KA is not only down-regulated in CKD but also correlates with several immune cell subsets, including Th17 and effector CD4+ T cells, implicating a role in immune cell differentiation or activation. Emerging studies have suggested that mutations in PI4KA lead to B cell metabolic dysfunction, mTOR pathway hyperactivation, and hypogammaglobulinemia—mechanistically linking PI4KA to immune dysregulation in chronic disease states (30, 31). Notably, PI4KA has also been associated with platelet activation and coagulation, underscoring its potential role in VTE pathogenesis (29). These mechanistic links between lipid signaling, immune modulation, and thrombosis provide a strong rationale for further investigation of PI4KA in CKD-VTE comorbidity.

Our ssGSEA results revealed distinct immune cell infiltration patterns in both CKD and VTE, modulated by the expression of the key genes HNRNPA0 and PI4KA. In CKD, higher infiltration of Th2 cells and immature B cells aligns with maladaptive immune responses contributing to renal fibrosis (32, 33). While Th2 cells are typically anti-inflammatory, their persistent activity drives tissue fibrosis through cytokines like IL-4 and IL-13 (34, 35). Additionally, a reduction in protective B cell subsets (B1 and B2 cells) has been linked to CKD progression, highlighting the importance of the B cell landscape in renal outcomes. The negative correlation between PI4KA and Th17 cells is noteworthy, as Th17 cells are potent drivers of kidney injury and fibrosis (36). This suggests that PI4KA may help regulate excessive Th17-mediated inflammation, potentially through pathways like TREM-2 in dendritic cells (37). The positive correlation of HNRNPA0 with effector memory CD8+ T cells and plasmacytoid dendritic cells further suggests its role in immune surveillance and memory responses within the kidney. In VTE, the immune landscape reflects sterile inflammation, with elevated levels of activated CD8+ T cells and effector memory CD4+ T cells, consistent with their roles in deep vein thrombosis. T cells influence thrombus resolution through antigen-independent activation and cytokine production, such as IFN-γ (38). Moreover, B cells can modulate thrombosis through cytokine-driven platelet production and by influencing T-cell responses (39). The association of PI4KA with immune cells, particularly its role in platelet signaling, links lipid kinase activity to immune cell function and thrombus formation. Overall, the immune infiltration patterns linked to HNRNPA0 and PI4KA not only correlate with disease outcomes but also reflect active immunological processes in CKD and VTE.

Our identification of 23 overlapping differentially expressed genes between CKD and VTE, including HNRNPA0 and PI4KA, supports the notion of shared molecular pathways underlying these seemingly distinct conditions. Both diseases exhibit systemic inflammation, endothelial dysfunction, and immune imbalance, which are likely driven by common upstream regulators. Through machine learning-based prioritization and robust validation, HNRNPA0 and PI4KA emerged as core hubs within these disease networks.

Furthermore, the nomogram model constructed from these genes demonstrated high diagnostic performance (AUC = 1.000), and its calibration and decision curve analysis indicated clinical utility. The non-invasive detection of these markers in PBMCs further enhances their translational potential, particularly in early-stage CKD or for assessing thrombotic risk.

## 5 Limitations and future perspectives

This study has several limitations. The qRT-PCR validation was conducted on a small cohort, and future studies should include larger, ethnically diverse populations to assess the generalizability of our findings. While we focused on HNRNPA0 and PI4KA, our analyses also identified 23 other overlapping genes that may play important roles in CKD and VTE. Investigating these genes further could provide deeper insights into immune regulation, kidney dysfunction, and thrombosis. Moreover, although we have suggested immune-regulatory roles for HNRNPA0 and PI4KA, direct mechanistic evidence remains lacking, and functional studies in immune and kidney cell models, or animal models, are needed. Future work should also consider integrating advanced modeling techniques, such as logistic frameworks with nested cross-validation, confidence intervals, and decision-curve analysis, to better validate the clinical applicability of our findings. Finally, ensuring feature selection and preprocessing within cross-validation folds will be essential to prevent data leakage and optimize model performance.

## 6 Conclusion

In conclusion, this study identifies HNRNPA0 and PI4KA as potential biomarkers and immune modulators in CKD and VTE through integrative multi-omics and single-cell analysis. Their consistent upregulation, strong diagnostic performance, and associations with immune pathways suggest important roles in chronic inflammation, vascular dysfunction, and immune-metabolic signaling. These findings offer a new perspective on the molecular overlap between kidney and thrombotic diseases and provide a basis for future therapeutic exploration.

## Data Availability

The original contributions presented in the study are included in the article/**Supplementary Material**. Further inquiries can be directed to the corresponding authors.
