# Supplementary material for: Differential gene expression profiling and machine learning-based discovery of key genetic markers in VTE and CKD
Source: Front Immunol. 2025 Oct 22;16:1654673. doi: 10.3389/fimmu.2025.1654673 (PMC12586094; doi:10.3389/fimmu.2025.1654673)
Supplement: Supplementary file 1 [file Table1.docx]

**Supplementary table 1. A list of primers used in this study.**

| Gene | Forward sequence (5’ to 3’) | Reverse sequence (5’ to 3’) |
| --- | --- | --- |
| GAPDH | GGAGCGAGATCCCTCCAAAAT | GGCTGTTGTCATACTTCTCATGG |
| PI4KA | CAGCTCTGACCAAGTGGAGAT | GCGGATGGTTGCATTTGGAA |
| HNRNPA0 | TGGCTTCGTGACCTACTCCAA | GGCCTCCGACAAAGAGCTT |
